# The IHI Rochester Report 2022 on Healthcare Informatics Research: Resuming After the CoViD-19

**DOI:** 10.1007/s41666-023-00126-5

**Published:** 2023-05-01

**Authors:** Carlo Combi, Julio C. Facelli, Peter Haddawy, John H. Holmes, Sabine Koch, Hongfang Liu, Jochen Meyer, Mor Peleg, Giuseppe Pozzi, Gregor Stiglic, Pierangelo Veltri, Christopher C. Yang

**Affiliations:** 1 University of Verona, Verona, Italy; 2 University of Utah, Salt Lake City, UT USA; 3 Mahidol University, Nakhon Pathom, Thailand; 4 University of Pennsylvania, Philadelphia, PA USA; 5 Karolinska Institutet, Stockholm, Sweden; 6 Mayo Clinic, Rochester, MN USA; 7 OFFIS, Oldenburg, Germany; 8 University of Haifa, Haifa, Israel; 9 Politecnico di Milano, Milan, Italy; 10 University of Maribor, Maribor, Slovenia; 11 University Magna Græcia, Catanzaro, Italy; 12 University of Calabria, Rende, Italy; 13 Drexel University, Philadelphia, PA USA

**Keywords:** Biomedical and health informatics, Artificial intelligence in medicine, Research trends, CoViD-19

## Abstract

In 2020, the CoViD-19 pandemic spread worldwide in an unexpected way and suddenly modified many life issues, including social habits, social relationships, teaching modalities, and more. Such changes were also observable in many different healthcare and medical contexts. Moreover, the CoViD-19 pandemic acted as a stress test for many research endeavors, and revealed some limitations, especially in contexts where research results had an immediate impact on the social and healthcare habits of millions of people. As a result, the research community is called to perform a deep analysis of the steps already taken, and to re-think steps for the near and far future to capitalize on the lessons learned due to the pandemic. In this direction, on June 09th–11th, 2022, a group of twelve healthcare informatics researchers met in Rochester, MN, USA. This meeting was initiated by the Institute for Healthcare Informatics—IHI, and hosted by the Mayo Clinic. The goal of the meeting was to discuss and propose a research agenda for biomedical and health informatics for the next decade, in light of the changes and the lessons learned from the CoViD-19 pandemic. This article reports the main topics discussed and the conclusions reached. The intended readers of this paper, besides the biomedical and health informatics research community, are all those stakeholders in academia, industry, and government, who could benefit from the new research findings in biomedical and health informatics research. Indeed, research directions and social and policy implications are the main focus of the research agenda we propose, according to three levels: the care of individuals, the healthcare system view, and the population view.

## Introduction

On June 09th–11th, 2022, a group of twelve health informatics researchers from academia and major research centers met in Rochester, MN, to assess a research agenda for biomedical and health informatics (BMHI) for the next decade. This meeting was modeled after similar meetings in related research areas [1, 2], and it was initiated by the Institute for Healthcare Informatics—IHI [3]. IHI is a non-profit professional organization and has the goal of connecting an interdisciplinary global community concerned with the application of novel approaches in computer, information, and data sciences, through suitable information and communication technologies, “to address problems in healthcare, public health, medicine, everyday wellness as well as the related social and ethical issues.” IHI is sponsoring the IEEE International Conference of Healthcare informatics (ICHI) where the authors met as a pre-conference event of ICHI 2022 in Rochester, MN.

The goal of the meeting was to discuss the current biomedical and health informatics research agenda in light of the changes and the lessons learned from the CoViD-19 pandemic and to report recommendations for a future biomedical and health informatics research agenda for enabling data-driven citizen-centered health and well-being. This document reports the discussed topics and directions for the future of biomedical and health informatics as result of the meeting.

Biomedical and health informatics [4] is the modern term for the field previously known as biomedical informatics [5]: “the interdisciplinary field that studies and pursues the effective uses of biomedical data, information, and knowledge for scientific inquiry, problem-solving and decision making, motivated by efforts to improve human health. Application areas range from bioinformatics to clinical and public health informatics and span the spectrum from the molecular to population levels of health and biomedicine.” Other terms, such as “computational medicine/health sciences,” “health data science,” “digital health sciences,” and “AI in medicine,” have been adopted to deal with similar/partially overlapping topics of the area [6, 7].

Research in biomedical and health informatics has been evolving for several decades and has achieved a level of maturity comparable to any modern biomedical science sub-discipline. Many high-quality journals [8] and conferences have become widespread, reaching worldwide diffusion.

In 2020, the CoViD-19 pandemic took the world by surprise, with threats and consequences that have been experienced only very rarely at such a global scale. This pandemic modified approaches, priorities, and social behaviors in multiple healthcare and medical contexts. The CoViD-19 pandemic tested, validated, and challenged some of the achievements of the research in our field. The pandemic also revealed some of the weaknesses and some of the uncovered areas of research that our field should consider in the incoming years. As a result, the research community is called to perform a deep analysis of the steps already taken, and to encourage more exploratory, more innovative, and long-haul work, focusing on the most promising and necessary directions to follow.

The literature already includes reports from a technical/computer science point of view about applications and approaches responding to CoViD-19-related challenges [9–16] as well as about the role of machine learning in controlling the pandemic from a clinical perspective [17, 18] and recommendations for evidence-based health informatics to combat the pandemic [19]. The current paper takes a BMHI perspective.

The remainder of this paper is organized as follows. Section 2 discusses the driving forces of the new research agenda; Section 3 proposes a research agenda for the next decade and Section 4 summarizes major the research directions. Finally, Section 5 finalizes some conclusions.

## Post-CoViD-19 Challenges and Opportunities

The major driving forces and underlying pillars that are driving the research agenda include phenomena that started before CoViD-19, but became more pervasive and potentiated since the pandemic. These phenomena, described below, are enablers for BMHI applications and research.

The data revolution allows us to infer from larger volumes and much faster and diverse data streams, information (summarization of data into population-level facts), knowledge—which applies the information to yield instructions and know-how, such as clinical recommendations and decision options, and finally wisdom—which incorporates ethical and aesthetic values and judgment to support making effective, efficient, and explainable decisions toward the clinical and societal goals [20].

Important movements have been shaping medical care in recent years. Among them, we mention:

i evidence-based medicine [21], which argues that clinical decisions need to be based on clinical evidence;
ii personalized medicine [22], which tailors healthcare to the individual patient based on their predicted response or risk of disease, often using predictive algorithms;
iii patient participation [23] and empowerment [24], which understands that the health of patients will improve when they take responsibility in becoming active and knowledgeable partners in shared clinical decision-making; and finally
iv the biopsychosocial model of health [25], which stresses the importance of dealing not only with physical disease but also with mental health and well-being.

According to these perspectives, some research directions in biomedical and health informatics are completely new, but most of them are well-known issues, which need to be addressed in an (at least partially) new way based on the CoViD-19 experience. Sections 2.1 describes existing phenomena that are driving forces; Sections 2.2, 2.3, 2.4, 2.5, 2.6 are focused on phenomena related to CoViD-19.

### The Data Revolution

In his book “Sapiens: A Brief History of Humankind,” Harari describes the revolutions of mankind. After the agricultural and scientific revolutions, we are now in the new age of the data revolution [26]. In this new age, human beings and society depend on data and on information technology (IT) in their daily lives. In the following, we report issues regarding data evolution w.r.t. life science:

i big data and ease of storage: Humans and organizations create data and leave their digital signatures everywhere, intentionally or as a by-product of daily work and life. In addition, data storage is very cheap and almost costless. We are collecting petabytes of new data, without the need to care about storage costs, though this may have deleterious energy and environmental effects. Data have variable formats, like alphanumeric (free text or coded/partially coded information, including semantic web), signal, image, video, or personal multimedia data [27]; new issues and concerns about data privacy arise [28].
ii maturing and effective artificial intelligence (AI)–based techniques for data analysis: Deep learning and transfer learning produced algorithms and tools that have revolutionized personal lives (e.g., Google translate, reverse image search) as well as clinical diagnosis. In the domain of radiology, some claim that trained professionals will be shortly replaced or highly aided by AI algorithms. Although this could be classified as an extremely revolutionary view of AI integration in healthcare, we believe that breakthrough discoveries in recent years have introduced many new challenges that will need to be addressed in the near future. In the case of radiologists, we might see solutions that will represent a mandatory toolkit for every radiologist to improve their performance, which could also mean that working without the support of AI would be considered non-compliant in some environments for the benefit of the patient [29]. This could be followed by new rules for many other healthcare specialists where AI has the potential to make a big difference in how they diagnose, predict, or do other related tasks, taking into account also the recently highlighted social fairness [30].
iii mobile health (mHealth): Data from wearable devices are helping people of all ages, genders, and geographical regions to connect, become educated, receive services, and live independently [31]. Smartphones are becoming the personal hub for health information management, and the usage of many health apps is free. However, there are also negative sides, including the lack of warranty and a poor regulatory framework for quality control of the wearables and the app itself. Lack of rigorous clinical trials that demonstrate the effectiveness of such apps, uncertainty about meaningful uses, and difficulty of integration of mHealth-collected data with Hospital Information Systems (HIS) and the Electronic Health Record (EHR) continue to be unresolved challenges [15, 16, 32].
iv Internet of Things/Internet of Healthcare Things (IoT/IoHT): Internet of Things/Internet of Healthcare Things, and the Ubiquitous Internet are collecting data automatically and continuously, in households, personal and professional environments. In addition, environmental sensor networks are providing continuous real-time data relevant to human health at low cost [33]. Integration of these data, with a great deal of time and spatial resolutions and qualities, into health decision-making, will remain a key research challenge for the foreseeable future.

### Prospective and Retrospective Data Collection for Learning

Historically, data requirements in health science research were defined by clinical researchers and informaticians prospectively, to support patient management, or to answer a research question. Furthermore, to generate clinical evidence, informatics and clinical research methods were defined. Obtaining evidence from data that is available but was collected for a different primary use is an avenue that has been explored over the last 50 years, and recognizes the great potential of big data availability as well as the need to study and address bias in data collection and data analysis [34]. During this quest, researchers need to explore ways in which the resulting knowledge, in the form of predictive machine-learning models, could make a difference in clinical care and could “translate to practical improvement in clinical processes or outcomes” [35].

Generating, collecting, and acquiring data has become easy, routine, and pervasive: wherever we turn, we discover devices that acquire and store data helping us to perform everyday tasks. The pillars of the data revolution, described in Section 2.1, made this possible at costs that become smaller each day, and their volume and resolution every day get larger and higher. Yet, the focus of the data, information, and knowledge was usually commercial and not necessarily related to well-being. Furthermore, most of that data is collected for different use and without a specific health-related purpose. From the pandemic, we have learned lessons on how we could collect, analyze, and use existing data and IT for improving health and well-being.

So, what has changed further during the pandemic and how can we leverage the change into a new informatics agenda for enabling data-driven citizen-centered health and wellbeing? This will be discussed in the following subsections.

### Data and IT as Essential Elements for Society and Individuals

As the pandemic emerged, it rapidly became evident that the only way to effectively contain it was to share data among individuals, hospitals, industry, and government at a national [36] and international level. Administration and individuals alike, in all sectors, including health, education, and labor, understood that we *must use* IT to cooperate and to control and mitigate CoViD-19. Also, because IT was effective in helping people to deal with the personal consequences of the pandemic, many people *wanted to use* IT. The most salient example was platforms that enabled people to remotely perform daily life activities (whether adults or school children) in the new reality that restricted movement and gatherings. Finally, organizations and governments, as well as many individuals, *wanted to obtain knowledge and explanations from data*, in order to be informed about the current state of the world, of their own personal state, about the near future, as well as being alerted about fake news spreading at an even higher speed than the virus did.

As the pandemic evolved, government and international healthcare agencies sought to provide the public with detailed and up-to-date information, including incidence and mortality statistics, prevention measures, vaccines, and treatments. An unprecedentedly large and broad segment of society was presented with information about a healthcare topic at an unprecedented volume and rate. This provided an opportunity to gauge public interest in such information, as well as the public’s capacity to absorb and process the information.

The public faced a number of challenges to making effective use of the information provided. The understanding of the virus and how it spreads evolved over time, which resulted in the public being presented with uncertain information and a frequently shifting picture. In addition, because the information was provided by a variety of sources, including healthcare agencies, websites, news outlets, and politicians, the public was often confronted with conflicting information and recommendations. This confusing picture was exacerbated by widely disseminated misinformation. One study found that as much as 75% of the sampled population in the USA reported being confronted with conflicting information about CoViD-19 [37]. In the words of WHO Director-General Tedros Adhanom Ghebreyesus, the world was fighting an “infodemic.” Some people were able to cope with this volume and shifting mix of information and reach informed decisions. Others suffered from information overload, resulting in poor prevention behavior decisions [38]. Correlations were also found between the amount of time people spent reading about CoViD-19 on social media and the incidence of anxiety and depression [39].

On the positive side, many citizens became more educated about the interpretation of information (and even data, to some extent) and about health, and realized the real value of data. Before the pandemic, most people realized the commercial value of data, like having their name, surname, and credit card number stored as cookies inside the browser of their smartphone to easily support purchases. People saw, on television and in the news, charts presenting data about the pandemic and visualizations of different key indicators over time; many became interested in the meaning that stems from the way in which the data was collected. For example, much of the public may now be aware of how viruses spread and they may understand the exponential spread of the virus. They may also understand that measuring the number of people with positive CoViD-19 tests depends on the sensitivity of the test that was used (home antigen test vs. PCR viral mRNA amplification test) and on the percentage of infected persons actually performing and recording the tests at medical centers. They understand that vaccines can fight against infection but also understand that viruses undergo mutations and changes that make the vaccines less effective and also that the immune systems of people vary and that older persons have weaker immune systems and are more at risk from the virus also due to comorbidities. They can see tradeoffs in measuring the number of infections vs. the number of severely ill CoViD-19 patients. However, the scientific community has not done a great job explaining that in these types of complex systems, there are always uncertainties and that science is constantly evolving, making new discoveries and reformulating prediction models.

### A Citizen-Centered Health and Wellbeing Care System

As a result of public interest in CoViD-19, many citizens have the appreciation and the need for attaining basic informatics and medical literacy to allow them to make correct inferences from data, information, and knowledge, and to act wisely. With increased awareness and literacy, many understood the value of data as well as personal initiative and hence are potentially willing to share their data, look at their data and interpret it, and act wisely upon it (i.e., react to events and rapidly map information into knowledge and processes, resulting in informed decision making). They want to make autonomous decisions about their behavior, exercising self-judgment that is informed by science but weighs in the trade-offs of their behavior. These tradeoffs account for physical and for mental health, which is compromised when they are in isolation. The everyday decisions that citizens make regard for example, when to visit elderly family members in uncertain situations, when to allow their children to attend a school trip, how many days a week to work from home vs. at their institution, and whether to attend an international conference—and what they could do in order to increase their own safety and that of others.

Data about health and the health of a single person are just a small brick in the wall that we need to build in order to protect ourselves against the pandemic and to build physical and mental resilience for future events. With the importance of health data being understood by all, the basic step in the direction of resilience building is—with no doubt—that of sharing data. The concept of *citizen-acquired* data is thus a key factor, and it embraces all the data collected by individuals as part of self-tracking and of a digital lifestyle, and which may be collected toward some primary goal, or incidentally. CoViD-19 has made it visible (but it’s not new) that users/citizens, their data, and their interaction with data are crucial for a new, citizen-centered healthcare system. The pandemic has also made it more apparent that there is a significant knowledge divide in modern societies that is exacerbated by political, religious, and social contexts that may disregard scientific facts and capitalize on science uncertainty to construct unfounded theories that rapidly propagate as misinformation.

### Telehealth and Virtual Care Systems

The advancement of technological innovation and digitalization in healthcare has brought tremendous opportunity in transforming healthcare by greatly expanding access to healthcare services at relatively low cost [40]. To lower costs, specialist care has been centralized with fewer but more specialized clinics whereas healthcare has been decentralized leading to a shift from in-hospital to primary care and advanced home care [41]. The resulting fragmentation of care calls for digital solutions, but adoption has been less than comprehensive.

The CoViD-19 pandemic has profoundly accelerated the use of digital health technologies where the integration of virtual and in-person care has been deployed widely across healthcare, demonstrating that it is possible to create a healthcare system that is more accessible, scalable, and sustainable. For instance, w.r.t. accessibility, it is indubitable that the future of healthcare will be more accessible which goes beyond virtual patient visits. Remote patient monitoring (RPM) systems have become very popular since CoViD-19 [42–44]. Advanced care at home with remote monitoring, and on-demand immediate medical care management, along with rapid response teams to deliver supplies and services that report to accessibility and remote services. Scalability regards remote diagnostic and monitoring options, and data integration processes (e.g., from electronic health records to remote diagnostics and AI). Finally, the healthcare systems should be able to allow sustainable services and more efficient partnerships among public and private providers. This may be able to transform healthcare into a platform for delivering patient-centric healthcare, where patients can seek care from a healthcare system anytime and anywhere and get support for self-management and prevention.

### Resilience and Agility

Health information systems play an important role in terms of the possibility of recovering from an emergency, not only strictly related to health systems but also related to social and working life. Data and context evolve rapidly and resilience models should be defined as able to react in terms of services for improving the quality of life for patients, with single healthcare systems, but also by governments.

The CoViD-19 pandemic also highlighted the importance of considering new kinds of information systems. Indeed, even by using cloud computing and social media, healthcare and medical information systems had to be merged and integrated within wider, often worldwide, information systems, aiming at supporting the (possibly) coordinated actions/decisions about safety policies, health, and medical nation-wide policies, worldwide travel restrictions, prevention of pandemic diffusion, teaching and education modalities, new working habits, and so on. We may define such a kind of information system supporting the activities of this “worldwide organization,” as a social information system.

Social information systems should be able to collect data that has to be quickly transformed or mapped into reliable and useful information. CoViD-19 tests, for instance, gave an example of collecting and transforming data into reliable information and knowledge to guide administrations in defining containment rules. This also implies:

i Motivational aspects (that are crucial for activating procedures, such as containment, vaccinations, and roles);
ii Cultural constraints to implement and obtain the same global effects; and
iii Reproducibility (replicate strategies/implementation/policy and measure outcomes in different contexts and regions). This is also related to scalability, discussed above. In the quest for resilience, we should recognize that in some countries (USA for example) there has been great concern about privacy issues [45].

Hence, there is a need to balance privacy preserving vs. public advantages.

The urgent need of addressing social and mental health, which incidence increased during CoViD-19 due to isolation, should be supported by a continuum of care involving different systems: education (e.g., school), social work and welfare, and the healthcare system. This calls for a vision in which educators and social workers—professionals and researchers—collaborate with healthcare professionals and researchers to address wellbeing in a holistic and translational way.

## The Agenda

The section discusses the major topics of the new research agenda, motivated by the driving forces presented in Section 2. To ease the reading, we group the topics into nine major areas.

### Prospective and Retrospective Data Collection

In times of crises, data collected incidentally (e.g., location data from which exposure and contact between a patient and other individuals may be inferred, or data from wearables that citizens donate to measure a population’s health [46]), though valuable, should be complemented by the collection of other prospective data, in which specific clinical questions and requirements are defined ahead of time. There is a need for semantic, technological, and legal approaches that allow the rapid and accurate collection of data that are needed to investigate and manage crises. Another issue that arose during the CoViD-19 pandemic concerns the timely collection and reporting of surveillance data, which is needed to effectively track disease spread and to provide input to population-based epidemiologic predictive models. This issue can be addressed through better data flow from clinics and through sensor networks or edge computing for environmental monitoring. Thus, in this regard, methodologies are required to be able to specify in a strong and reproducible way the process for data collection. This process should continually check and validate data collection, so that data are trustworthy within the expected uncertainty boundaries that characterize any measurement [47], and the results based on such data are reproducible within well-specified tolerances [48, 49]. As far as we know, the monitoring related to CoViD-19 is probably the first time that data were provided daily at a worldwide scale, to both understand how a disease was evolving and to try to predict its evolution. Haendel et al. [50] demonstrated that in case of emergency, collaborations can form quickly and data can be integrated at an unprecedented speed and magnitude, as in the case of the USA National CoViD-19 Cohort Collaborative (N3C) [50]. An international effort at rapid data collection and integration from the electronic health records of over 350 hospitals in eight countries was created in early 2020, during the early stages of the pandemic [51].

Related to data collection, we pose the following specific research challenges (see Table 1).

i At the individual level, can we harness the power of mass data (“patients like me”) to answer questions that an individual is not even aware of, but are important for him/her?
ii At the healthcare system level, can we develop novel methods for data collection that quickly and precisely collect from citizens the needed prospective data necessary to manage the crisis?
iii How can we accelerate the acquisition of data relevant to public health decisions?

**Table 1:** New research topics and policy implications, arranged according to the three views of the individual, the healthcare system, and the population

View	Research questions	Policy implications
Individual	∙ (see Section 3.1) Harnessing the power of mass data (“patients like me”) to answer questions that an individual is not even aware of, but are important for him/her. ∙ (see Section 3.2). Methods and tools to enable citizens to curate their data to add meaning and context. ∙ (see Section 3.3). Methods for detecting and mitigating legal issues of contradicting rights: own your data and use it to support yourself vs. right to protect the public by mandating data sharing. ∙ (see Section 3.6). Explanation and education methods and tools that can help persons to make sense of their data. ∙ (see Section 3.8). Lifelong health support systems that reflect phases of changes in the person’s well-being (where purposeful tracking with decision support and behavior-change interventions is desired) and times of relative stability (with merely incidental tracking). Systems that increase resilience and proactively engage the people at risk. When the social surrounding is not available/not functioning, a technical system is at least the second-best choice.	∙ (see Section 3.3). The right to access vendor-specific data from wearables and sensors of an individual and online activities in a standardized way that allows the data to be analyzed and integrated into the personal EHR; Technical specifications must balance the completeness of standards (e.g., HL7, FHIR) with vendor needs for simple interfaces. Regulations should allow individuals related to control their data (e.g., European Health Data Space Regulation (EHDS) [55], Health-X [27]) and could provide access to parts of the data in a federated way so individuals could compare their health status with a subpopulation using aggregated data to ensure data privacy and confidentiality. “The right for usability”: every individual must be able to understand what a specific technical system does and what consequences taking or not taking a digital activity has. Health record banks [67] can provide services for integrating and accessing clinical and genomic data into patient-centric longitudinal and cross-institutional health records. The patient data will be captured comprehensively and consistently with legal/policy and privacy-preserving techniques. Health record bank consultants can recommend the level of “insurance” that is good for an individual. Health record bank services could obtain the recommendations from different ML models and say which program they recommend for the given individual (e.g., what diet he/she should follow, based on his/her microbiome).
Healthcare system	∙ (see Section 3.1). Data collection methods that quickly and precisely collect from citizens the needed prospective data necessary to manage the crisis. ∙ (see Section 3.2). Methodologies to interpret this (retrospective) data that is different from traditional (prospective/purposeful) medical data. ∙ (see Section 3.6). Methods converting the patient’s self-tracked data into a summary or visualization that is meaningful for the medical actors (doctors) and integrating it with the patient’s EHR.	∙ (see Section 3.2). Usable interfaces, similar to that of PubMed search, allow accessing shared ML models and shared datasets; these may be used by genetic consultants or endocrinologists, radiologists, neurologists, and other professionals. ∙ (see Section 3.5). Methods to create a fair healthcare system, and to characterize fairly the subgroups affected by a pandemic or other health crisis. Legislation should be established to define these requirements.
	∙ (see Section 3.7). Methods to allow organizations to screen the quality of the information they provide to the AI model to avoid misinformation. ML model should alert that human inspection is needed if the patient for which DSS is provided is not well represented in the dataset. ∙ (see Section 3.8). Risk calculators based on validated ML models.	∙ (see Section 3.7). Regulation on evaluation of ML models like Transparent reporting of a multivariable prediction model for individual prognosis (TRIPOD) [97] or diagnosis or Software as a medical device [98]. ∙ (see Section 3.8). Acts, technology, and standards for continuity of care could allow clinicians to use tools that can obtain evidence-based advice combining clinical guidelines and predictive models. Legislation is needed to allow accessing and defining inclusion and exclusion criteria for the “other” similar population, which can come from the same healthcare organization, but potentially also from national or international shared data.
Population	∙ (see Section 3.2). Design models are able to gather and integrate information from different and heterogeneous prospective biomedical research studies with citizen-acquired data. Methods for adding context and environmental data be added to EHR data. ∙ (see Section 3.6). Methods for analyzing citizen-generated data, considering biases. ∙ (see Section 3.8). Methods that can help governments and local governance agencies to establish policies based on AI models developed using citizen-acquired datasets. Educators, physicians, psychologists, and health managers, can together define requirements for data collection from the education, social, and healthcare systems and from citizens, as well as define the purpose of ML models and evaluate these models. ∙ Temporal Prediction Frameworks (see Section 3.4). The inherent timing of what is being predicted and the ability of the prediction models to consider that timing: any prediction is meaningful only with respect to the time needed to observe the outcomes of the actions taken.	∙ (see Section 3.1). Methods for accelerating the acquisition of data relevant to public health decisions. ∙ (see Section 3.2). Legal policies and regulations for exchanging, sharing, and receiving data for research (research done by research institutions in academia, public sector, industry, and government) at an integrated national and international level. ∙ (see Section 3.3). Technological tools for allowing sharing of data in a secure, privacy conserving yet meaningful way (e.g., encryption, de-identification). European Health Data Space (EHDS) [55] regulations that offer the possibility or mandate of sharing ML models and data. Sharing via simulated population data that has the same properties as the data of your organization. ∙ (see Section 3.7). National health services could take a ML model from an organization that developed it on a smaller dataset and will evaluate it on a national dataset, and then move it to an international level. Regulations mandating organizations that release information or knowledge to screen it to see that it isn’t fake. ∙ (see Section 3.9). Approaches for the curation of the models will clarify what approaches, algorithms, and models will still be usable in the future after the environment changes due to immunization and the evolution of the CoViD-19 virus.

### Data Sharing and Integration

Gathering enough data to represent all classes of patients in a dataset that serves as the training set for a machine learning (ML) model is crucial, and could potentially be done via data sharing for clinical applications. We speculate that when we face future crises, individuals would be interested in sharing daily-life data for research without having specific clinical questions defined ahead of time (prospectively). Such data could be used retrospectively to collect evidence regarding effective interventions from the large quantity of population data shared by individuals. An example is the German Corona data donation app with currently around 190.000 monthly active donors [46]. Considering that evidence has been so far collected mainly by prospective studies, novel data integration methods would need to be developed to allow unbiased and statistically sound integration of retrospectively secondary use data with prospectively collected data; even without integration, simply interpreting retrospective data and generating proper evidence from it is not easy. An example of such a study can be found in [52], where a retrospective approach was developed to evaluate potential adverse outcomes associated with delay of procedures for cardiovascular and cancer-related diagnoses, in the context of CoViD-19.

The pandemic highlighted the urgency for data integration, particularly in epidemiologic predictive models. Indeed, data must often be integrated from a variety of sources that influence the spread of the virus, including:

i the virus properties;
ii the properties of the environment in which the virus is spreading; and
iii the human behavior by different social groups within the environment which dictates exposure [53].

Data exist in medical institutions, or are collected at a national level by the government—from medical groups and from individuals (e.g., via self-reporting apps). Since many groups may be working with the same national datasets, data integration should not be done anew for each ML model that is developed. Integration of data from different national healthcare environments can be complicated by the adoption of different standards in each national context. Initiatives such as the European GAIA-X infrastructure project [54] may provide the technical foundation for such integrations. In combination with the European Health Data Space regulation [55], new opportunities are currently being made possible and explored, e.g., by the German “Health–X” project [56]. CoViD-19 is only one of the prominent examples where data integration from different regional and national healthcare and clinical systems is required to provide support to achieve effective solutions [57]. Other examples come from other infectious diseases [58], environmentally sustainable development, biodiversity, and climate change monitoring and evaluation [59–62]. Another example of data sharing and integration is between government ministries responsible for human health, animal health, and the environment in order to better detect and control the spread of zoonoses [63]. However, not all countries have the infrastructure or legal framework needed for such data sharing and integration on a national and international level.

Data integration methods should allow the integrating of different types of data, like EHR data and data shared by citizens, coming from sensors, as well as self-reported in structured forms, free text, wave, or image formats. Many challenges exist in transferring the integrated data to an EHR system, including syntactic and semantic issues as well as legal and ownership regulations. This requires a framework and standards to support and encourage organizations to share their data. Considering public health, the CoViD-19 scenario confirms our arguments: during the pandemic, cross-border sharing of epidemiologic data became essential to track the transnational spread and inform control measures [64]. While less dramatic, this is also the case with other infectious diseases [65]. Additional efforts should be made at the global and local levels to encourage data collection and reduce the fraction of missing data. The impact of missing data on the effectiveness of the solutions built on these data is significant [66]. In order to guarantee that electronic health record data is optimally used for patient and public benefit, government and professional organizations must prioritize efforts, like the following: (i) providing lifelong learning opportunities for healthcare experts to address these limitations; (ii) informing the general public, whose support is critical, about the social advantages of properly sharing data.

Data federation is an approach for data integration that offers a means of querying and analyzing information from multiple systems as if it all resides within a single, harmonized data store, without consolidating the data into a single store. Health record banks [67] could provide services for integrating and accessing clinical and genomic data into patient-centric longitudinal and cross-institutional health records. Consultants can recommend the level of “insurance” that is good for the patient. They could obtain the recommendations from different ML models and say which program they recommend for you (e.g., what diet you should follow, based on your microbiome). This will allow patient data to be captured comprehensively and consistently with legal/policy and techniques for privacy-preserving techniques. Such multimodal data fusion techniques are being developed [68] but there are still many questions to be answered. In which contexts should data be integrated? When is data federation appropriate? When is early or late data fusion indicated for features feeding the ML model?

Related to data sharing and integration, we pose the following research directions (see Table 1).

i At the individual’s level, how can the citizens curate their data to add meaning and context?
ii At the healthcare system level, can we develop methodologies to interpret retrospective data available in an institutional EHR, which is different from traditional (prospective/purposeful) medical data?
iii At the population/research level, we offer several specific research directions: can we design and define new models able to gather and integrate information from different and heterogeneous prospective biomedical research studies with citizen-acquired data?
iv How can context and environmental data be added to the data? As an example, social and behavioral determinants of health (SDoH) [69] play a key role. Social determinants of health (SDoH) are the conditions in the environments where people are born, live, learn, work, play, worship, and age that affect a wide range of health, functioning, and quality-of-life outcomes and risks: major issues are on economic stability, education access and quality, healthcare access and quality, neighborhood and built environment, social and community context. Some more facets need to be considered to better focus the entire environmental data landscape.

We also pose the following policy implications questions (see Table 1).

i At the individual’s view, can we develop data collection methods that quickly and precisely collect the needed prospective data from citizens that are necessary to manage the crisis?
ii At the healthcare system view, can we develop usable interfaces, similar to that of PubMed search, to allow accessing shared ML models and shared data sets; these may be used by genetic consultants or endocrinologists, radiologists, neurologists, and other professionals?
iii At the population/research view, what legal policies and regulations should be developed for exchanging, sharing, and receiving data for research (research done by research institutions in academia, public sector, industry, and government) at an integrated national and international level?

### Data and Model Privacy

The need of sharing and analyzing large amounts of data coming from a wide population highlighted the further issue of health data privacy [28]. Data privacy, especially related to the healthcare domain, was already a discussed topic before the CoViD-19 pandemic and was also the main focus of recent laws (see, for example, the recent GDPR [70]). “In light of recent changes in technology, applications, social media, and other platforms, and the increasing generation, collection, use, sharing, and selling of personal health information,” the USA Senate introduced in 2022 the Health Data Use and Privacy Commission Act. This act is intended to modernize the Health Insurance Portability and Accountability Act of 1996 (HIPAA) and account for emerging healthcare technologies [71]. This represents a shift of paradigm from “data privacy” to “data use privacy,” which is the current approach in the USA for genomic data [72].

It is now even more evident that it is necessary to also consider the overhead for conducting research if the right to privacy is perceived in isolation and without considering in a holistic way all the social and healthcare consequences. Indeed, there is the need to strike a balance of data privacy vs. public interest, which is both from a long-term perspective (basic research) and a short-term perspective (applied research to forecast and manage pandemic events). From a user perspective, a—possibly only perceived—lack of proper privacy may considerably hinder the acceptance of digital health solutions, as shown by the discussion around the German Corona warning app that, although probably a prototypical solution from a privacy point of view, still received considerable mistrust and refusal.

A technology that may potentially help in reestablishing trust is blockchain [73]. Blockchain technology uses a decentralized and distributed environment without central authority; entries are simultaneously secure and trustworthy when using state-of-the-art cryptographic principles. While blockchain is widely used in cryptocurrencies, its use in health informatics has been very limited. The readers are referred to for reviews [74, 75], and examples of blockchain applications in healthcare [76, 77].

As an example, federated learning in healthcare can get profitable advantages from blockchain technology: federated machine learning (ML) may run algorithms over data that are neither shared in full nor locally stored, thus easing the privacy issues which arise when dealing with healthcare data [78, 79].

Related to the research agenda, we pose the following research questions (see Table 1).

i At the individual’s level, can we develop methods for detecting and mitigating legal issues of rights that may contradict? The right to own your data and the right to protect the public may interact. An individual may wish to provide his/her data to receive recommendations for him/herself but not to share the data with others.
ii At the population/research level, can we develop technological tools that would allow sharing data in a secure, privacy-conserving yet meaningful way (e.g., encryption, de-identification), balancing between the right for privacy and the quest to improve the ways in which we fight pandemics?

### Temporal Prediction Frameworks

The importance of *temporal prediction frameworks* for guiding national and organizational policies and regulations for fighting the pandemic was essential; governments, school systems, and multiple community organizations were informed by predictive models to determine and implement policies relating to mitigation, such as limitations on gatherings, masking, distancing, and lockdowns, as well as to prevention via vaccination. An important and often underestimated aspect of the predictive models was the inherent timing of what was being modeled and the ability of the models to consider that timing. As an example, the prediction of CoViD-19 spread is meaningful only with respect to the time needed to observe the outcomes of the actions taken. Indeed, in a short period, it is well-known that lockdown measures will not have measurable effects immediately [80]. In addition, lags in data reporting can have a significant impact on the accuracy of predictive models. This has been studied, for example, in the case of real-time dengue prediction [81].

In our new research agenda, we need to ask ourselves, related to the population/research level (see Table 1): what new questions could we answer with longitudinal data, and can we invent new AI methods to answer them?

### Quality of Data and of ML Models Developed from Datasets

The pandemic underlined the issues related to the *quality of data*. Data quality and completeness is a multi-faceted issue: it has to be considered both at model construction time and at inference time. Indeed, even though the model has been defined by using high-quality data, at inference time, a model may perform significantly worse than in testing during construction due to missing data or poor-quality data. This can be further complicated by the fact that user-generated data may have originally been collected for entirely different purposes and is now re-purposed for different use. Such a situation, long well-known, is becoming even more evident with pandemic data when decisions potentially impacting a large part of the population are based also on data having a partially known and controlled acquisition and transformation process. New concepts based on the idea of “fit for purpose” may be useful in this area as different types of studies may need different data quality characteristics, making the concept of absolute data quality less desirable [82, 83].

Uncertain and incomplete data caused many false positive cases in CoViD-19 contact-tracing systems [84]. In Israel, a collaboration between the Ministry of Health and the domestic security agency, conducted widespread tracking and relied on a classified database that has existed for 18 years, did not rely on informed consent, but was much more effective [84]; it increased the sensitivity and specificity of contact tracing and was accepted by public opinion as necessary. However, such an approach is not expected to be adopted universally, considering political climates and strong anti-establishment sentiments in a substantial portion of the population.

Because ML models are created from data that may not be complete, the model may have issues with *generalizability* and *transferability*. Model accuracy may be different for different clinical settings or for different geographic regions beyond those from which the original data came. A well-known classical example is that of the Leeds abdominal pain diagnostic system, developed by de Dombal and colleagues in the early 1970s, was very reliable for the local population but did not reach the same accuracy elsewhere [85, 86]. In the USA and other countries without a national health care system, this is especially problematic for underserved populations that receive care in settings in which informatics infrastructure and the use of electronic health records are suboptimal. This can make it difficult to aggregate data at the national level [87–89].

Considering the social changes induced by the pandemic, it has been widely recognized that the CoViD-19 pandemic caused a further distancing in many different contexts (between developed and developing countries, between groups of different ethnicity in a single country, between people with different incomes and different levels of education.) For data analytics, it means different demographic groups may not be equally represented in the predictive models. An important question is how to guarantee and measure fairness? Indeed, poor accuracy can arise because of groups underrepresented in the data and groups with limited positive cases. Moreover, different demographic groups may need different sets of attributes for allowing the proposed models to reach results of acceptable quality. “Fair AI” has drawn significant attention in recent years [30]. Fairness needs to start from the data collection stage, collecting data from all subgroups of the population [30]. Algorithmic approaches for preventing or correcting bias can be done at the pre-processing, in-processing, and post-processing stages [90]. The pre-processing approach performs data transformation to reduce biases or discrimination of the training data by mitigating the sensitive variables. The transformation may include removing attributes that are highly correlated with the sensitive attribute (suppression), assigning weights to the tuples in the training dataset (reweighting), and stratified sampling [91]. The in-processing approach intends to develop an optimized model that maximizes fairness and performance. The post-processing approach applies a transformation to model output to improve prediction fairness. Several metrics have been identified to measure fairness. For example, statistical demographic parity defines fairness as an equal probability of the predicted class for all demographic groups and such metric requires the predicted class independent of the demographic groups [92].

Shall we be able to apply fairness methods effectively to create a fair healthcare system, and to characterize fairly the subgroups affected by a pandemic or other health crisis? The legislation would be established to define these requirements.

### Explainable and Responsible AI

Historically, communication among care teams was based on language, and in the clinical setting was information-dense. When healthcare delivery becomes data-intensive, it requires explainable and interpretable AI to assist decision-making by healthcare professionals as well as by patients or citizens, which requires explanations at different levels of health literacy and information needs. For example, practitioners require explanations of recommendations from clinical AI–based decision support systems in order to obviate the “black box” problem, in which the reason for a recommendation is either opaque or missing. On the other hand, the lay user of a medical recommendation system may need explanations that are simpler, do not require clinical knowledge, and are consonant with the user’s health and general literacy level. In both cases, explainability leads to trust in what the system is telling the user.

*Explainable* AI (XAI) is currently an important multidisciplinary research topic in biomedical and health informatics, bringing in computer science, bioethics, and implementation science researchers more broadly. At the same time, foundational research efforts are still needed to go beyond the presentation of features and their positive or negative contribution, as facilitated by SHAP (SHapley Additive exPlanations) [93]. Holmes et al. highlight and focus on the main related concepts and technical aspects underlying such term XAI [70]. XAI in medicine requires considering the meaning and the relationships among terms such as interpretability, understandability, but also usability, and usefulness. XAI has to be related to data and to their quality, in order to allow stakeholders to understand why the AI-based systems are providing some (possibly partially unexpected) results. Most of the current work on XAI is concerned with generating explanations of inferences from deep learning models since these are the best-performing models for large classes of problems. Techniques focus on identifying the important features of the predictive model by saliency maps or generating post hoc explanations by approximating the complex and opaque models [94]. These techniques are helpful to artificial intelligence and machine learning scientists in enhancing their models but not necessarily offering a meaningful explanation to health professionals. At the same time, techniques such as Bayesian networks use knowledge representations that are inherently explainable. Unfortunately, for many problems, e.g., medical diagnostic image interpretation, Bayes nets are not the best performing technique. A promising recent approach, applied in radiology, combines deep learning models with Bayesian networks, using the deep learning model for feature recognition and the Bayes net for differential diagnosis generation [95]. Similarly, DeepProbLog integrates probabilistic logic programming with deep learning models by adding neural predicates for feature extraction [96].

On the other end, XAI has to be heavily connected to the final users (i.e., stakeholders), be they nurses, physicians, public health officers, or lay citizens. Indeed, both the way and the concepts used in explanations have to be finely tuned with respect to the role and the background of the expected user. During CoViD-19, citizens increased their consumption of health data and the results of analyses. However, a variety of information was presented to citizens in a variety of formats. It then becomes a challenge for citizens to be able to draw conclusions concerning risk and implications for their own behavior. Explainability becomes an important issue here both in terms of understanding the information and in terms of trust in the information.

The process of explanation is socio-cognitive. The cognitive process determines an explanation with sufficient information for a given event and the social process is transferring knowledge between the AI systems and the users through interaction. Continuous interaction is important given the initial explanations of the prediction, it facilitates further interrogation with user-driven questions by the healthcare professionals. There are two types of explanations, information-based explanation and instance-based clarification [94]. The information-based explanation can be extracted from the documentation of the implemented predictive model to address questions related to input, output, process, and performance. However, the instance-based clarification will need to be generated by examining the instances through executing the predictive model to address questions such as why, why not, and what if. For example, users may want to know what the prediction model may recommend if certain parameters of an instance are changed or how much the parameters are changed to change the prediction results. Can education help non-technical people consider results and interpret them relative to their own personal contexts?

Furthermore, we pose some research questions related to explainable and responsible AI (see Table 1).

i At the individual’s level, we ask, can we invent novel explanations and education methods and tools that can help persons to make sense of their data?
ii At the healthcare system level, we ask, can we develop methods that could convert the patient’s self-tracked data into a summary or visualization that is meaningful for the medical actors (doctors) and integrate it with the patients’ EHR ?
iii At the population level, we ask, can we develop methods for the analysis of the new citizen-generated data, considering biases?

### Quality Assessment of AI Models

Data quality, model performances, and explainability—together with the need of introducing AI-based data analytics for clustering, prediction, and decision support in real clinical settings—push for having quality assessment procedures for AI software tools. Regulations should be put into place for evaluating and reporting on AI-based decision-support systems, such as Transparent reporting of a multivariable prediction model for individual prognosis (TRIPOD) [97] or the machine learning (AI/ML)–based software as a medical device (SaMD) proposal [98]. The final goal would be having a reproducible pipeline of experiments witnessing the correct behavior of the software tools in controlled and verified conditions, with some progressive steps, similarly to the preclinical and clinical phases of drug trials, followed by an authorized certification, and by a post-marketing monitoring [99]. It is important to note that ML models work well on the majority of cases but may not work well for some populations that are underrepresented or may even have a systematic bias towards certain populations. For example, Obermeyer [100] finds a racial bias in an AI algorithm that is widely used in the USA, reducing the number of black patients in need of care by half, compared to white patients. In such cases, the ML models should alert that human inspection is needed (e.g., the radiologist should inspect the radiology report because the AI determined that the conclusion is not justified by the findings or because the patient is different than the population on which the ML model was trained).

The regulatory framework by the USA FDA for machine learning applications is still evolving. The FDA has followed the well-established principle that if the machine learning application is used by a provider and is mediated (i.e., does not trigger an automatic intervention), then the FDA does not have to regulate the application. But there have been considerable discussions arguing both in favor and against a much higher level of regulatory framework [98, 101–104].

Education also plays an important role here. The ease of applying powerful ML approaches to medical data means that technical people are often creating ML models without fully understanding the issues and limitations (e.g., potential biases) in producing reliable models. As researchers, but also as teachers, we must teach people about responsible AI and potential pitfalls in applying AI techniques.

Educators, physicians, and psychologists, and health managers, can work together to define requirements for data collection from the education, social, and healthcare systems and from citizens, as well as define the purpose of ML models and evaluate these models—especially for mental health management, applications, support, and prevention via mobile health (mindfulness, positive psychology, isolation, and social impact).

Another very important aspect of ML applications in medicine is the development of strong frameworks to evaluate the accuracy of individual predictions and not the commonly reported merit functions like AUC (Area Under the ROC Curve) or predictive values for entire populations. To accomplish this, we advocate looking into other fields and adapting well-established methods like end-to-end uncertainty quantification [105, 106]. A review on how to use conformal prediction for this purpose has been published recently by one of the authors [107].

Two research questions, at the healthcare system level, that our community should be answering related to quality assessment of AI models are (see Table 1):

i Can we develop practical methods to allow organizations to screen the quality of the information they provide to the AI model to avoid misinformation (ethics)?
  ML models work well on the majority of cases but may not work well for some populations that are underrepresented. In this case, the ML model should alert that human inspection is needed (e.g., the radiologist should inspect the radiology report because the AI determined that the conclusion is not justified by the findings).
ii Can we develop methods and tools to support this?

Finally, a policy implication at the population level is that national health services could take a ML model from an organization that developed it on a smaller dataset and will evaluate it on a national dataset, and then move it to an international level.

### Learning Health System

The new challenges regard the evolution of health informatics structures and systems to be able to react to emergency conditions, at institutional, national, and international levels. Indeed, in the last 2 years, since the beginning of 2020, health administration and services changed their approaches to health informatics systems, moving from a mechanism of supporting health systems in data management and storing, to mechanisms able to anticipate and predict event evolution (e.g., prediction of pandemic evolution and diffusions, as well as simulation of vaccines and virus evolution).

**Fig. 1:**
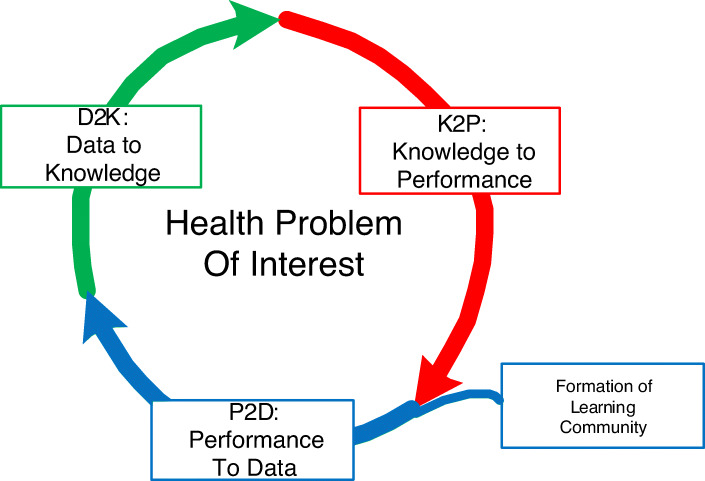
Learning Health System (LHS) cycle model from [108]

Addressing urgent needs for the CoViD-19 pandemic builds the foundation for how to plan the future for care delivery with a nimbler, patient-centered digital healthcare system that can better stand up to future challenges. During the CoViD-19 pandemic, the need for agile and resilient clinical and research information systems became rapidly manifest. One approach to address this need was the Learning Health System (LHS) model [108], which provides a conceptual framework for identifying and leveraging information and organizational resources in the context of a pipeline that has a repetitive, cyclic structure, as shown in Fig. 1.

The LHS and variations thereof have been particularly important as frameworks for mounting informatics-centric responses to the CoViD-19 pandemic. For example, an LHS model was adopted and adapted at the University of Alabama at Birmingham to enhance resilience and a proactive response to the pandemic [109]. Through the course of developing this model, the investigators identified seven contributing organizational components of their health system and academic center that were critical to achieving effective responses; informatics capabilities figured prominently in this model, involving informatics and information technology groups that partnered to leverage the substantial information resources at the institution. Another example is the system established at Assistance Publique-Hôpitaux de Paris (AP-HP), a public health hospital that instantiated the Health Data Space (HDS) as an extension to its clinical data warehouse in response to the pandemic [12]. The HDS is described as a “key facilitator for data-driven evidence generation and making the health system more efficient.” While the LHS provides a robust model for learning from clinical data, relatively few reports of a successful learning pipeline have been reported. Dash et al. have created such a pipeline that could be effective in responding in an agile and rapid way to events like the CoViD-19 pandemic [110]. Payne et al. make a strong case for the integration of informatics and healthcare IT in establishing a robust LHS for coordinating the surveillance of and response to the CoViD-19 pandemic and other public health emergencies [111].

In addition to LHS approaches, others have created networks that facilitate resilient and rapid responses to public health emergencies such as the CoViD-19 pandemic. For example, Duchen et al. report on a system that includes neighborhood-level data for tracking CoViD-19 vaccine uptake [112]. An extensive clinical information network was established in Kenya, focusing on 22 pediatric hospitals that have been used for CoViD-19 surveillance [113]. Although this network was not set up as an LHS , it provides the basic infrastructure for developing one. Vahidy’s work on developing a retrospective research task force is an excellent example of how the LHS model can be adapted and exploited to support rapid observational research using clinical and other data [114].

An LHS could provide decision support for increasing resilience and health literacy. What are some new opportunities for interventions or applications building on top of a person’s own health data, that are inspired by the public’s interest in resilience-supporting health and wellbeing?

As a first example, we envision a lifelong health support system accompanying and supporting every citizen throughout their whole life. Such a system would integrate seamlessly and unobtrusively with daily life. It would be based on data incidentally collected from wearables, IoT, health records, and the digital traces that we all leave as part of our digital lives. In times of stability, when everything is fine, the system would be mostly silent, being there merely upon request. During phases of changes in the person’s life well-being, such as when entering a new stage of life, the system may become active, offering decision support and behavior-change interventions. Changes in health, which may go slow and undetected, may be identified early by such a system, and warnings and recommendations may be issued, encouraging a more intense, purposeful interaction to help find reasons for changes and take appropriate measures.

A second example is to rely on AI-based systems that increase resilience and proactively engage people at risk of mental and social problems. When the social surrounding is not available or not functioning, a technical system is at least the second-best choice [115–117].

Finally, once reliable datasets and models used in a LHS have been created, they need to be curated for further reuse. Curation of the datasets and ML models should consider what approaches and algorithms will still be usable in the future, after the environment would change, for example, due to immunization and the evolution of the CoViD-19 virus. Note that data acquisition is becoming less expensive, but the cost of data curation which is still done very much by skilled humans is very costly. More research in autonoetic annotation and scalable and reproducible concept extractions is needed.

Table 1 presents some research questions that we pose to our community. At the individual’s level, we propose two specific ideas for new opportunities for interventions/applications building on top of a person’s own health data:

i lifelong health support systems that reflect phases of changes in the person’s wellbeing (where purposeful tracking with decision support and behavior-change interventions is desired) and times of relative stability (with merely incidental tracking);
ii systems that increase resilience and proactively engage the people at risk. When the social surrounding is not available/not functioning, a technical system is at least the second-best choice.

A challenge at the healthcare system level is to develop acts, technology, and standards for continuity of care. These could allow clinicians to use tools to obtain evidence-based advices based on traditional clinical guidelines and on predictive models; such models are based on data from patients similar to the patient being taken care of. Changes in legislation are needed to allow accessing and defining inclusion and exclusion criteria for the “other” similar population, which can come from the same healthcare organization, but potentially also from national or international shared data.

Finally, at the population level, we ask:

i what questions can be answered with citizen-acquired data, and what are the new opportunities (with longitudinal data) that can help governments and local governance agencies to establish policies based on AI models developed using citizen-acquired datasets?

### Digital Disparity and Trust

As a discipline, biomedical and health informatics is at the interface of technology, people, and process where the implementation and dissemination of informatics solutions depend on our ability to formulate the solutions through a social, ethical, and trustworthy framework.

Health disparities, referring to preventable differences in the burden of disease, injury, violence, or opportunities to achieve optimal health, have been experienced by socially disadvantaged populations. Even though various policy efforts are aimed at reducing health disparities, evidence mounts that population-level gaps in healthcare quality are increasing [118]. This widening of disparities is likely to worsen over the coming years due, in part, to our increasing reliance on Internet-based technologies to disseminate health information and services. For example, even though telehealth has been considered a way to close the healthcare gap between the rural and marginalized urban populations, the CoViD-19 pandemic has surfaced the disparity in the access to technology, i.e., the digital divide as a social determinant of health [16, 119].

Research has shown that the benefits of advanced medical technologies disparately benefit people belonging to different demographic groups. For example, in the USA people who identify as Black or African American have been shown to receive less benefit from an AI algorithm, which uses claims data as surrogates for resource needs and tends to identify people in need of extra health resources [100]. Meanwhile, they also suffer more hidden hypoxemia due to inaccurate non-invasive pulse oximetry measurements [120].

There are opportunities as well as challenges in leveraging digital innovations to address health disparities. It is critical to gain the trust of people in improving the data capture of social determinants of health information, generating evidence-based interventions, and implementing them in the practice of healthcare aiming for optimizing health outcomes for those socially disadvantaged populations. More research is needed to identify important social determinants of health, variables needed to be collected from patients, how to generate better data-gathering approaches that mitigate cultural or socioeconomic differences in reporting, and how to engage patients and communities to generate and deliver fair, inclusive, and trustworthy digital innovations. To move the field forward, in June 2021, World Health Organization (WHO) published a guidance on Ethics and Governance of Artificial Intelligence for Health, which acknowledges that digital solutions hold great promise to improve diagnosis, treatment, health research, and drug development and to support governments carrying out public health functions, including surveillance and outbreak response but must put ethics and human rights at the heart of their design, deployment, and use [63].

Trustworthy in AI is strongly connected to policy-making based on social, ethical, and trustworthy frameworks. In this regard, curation of the AI models is important. This curation process should consider the following question (see Table 1): what approaches, algorithms, and models will still be usable, equitable, and trusted in the future after the environment changes due to immunization and evolution of the CoViD-19 virus?

## Research Directions and Discussion

We discussed above the main directions and issues learned from the CoViD-19 pandemic, vis-à-vis health informatics. We report in Table 1 a summary of such issues as the results of the Rochester meeting.

Research directions and social and policy implications are the main objectives of the newly proposed research agenda. These can be viewed on three levels:

i the care of individuals;
ii the healthcare system view, which provides patient-centric medical care for sets by clinicians and by clinical institutions, customized for people with similar characteristics and needs; and
iii the population view, where research aspects and policy have to be considered in a large scale perspective.

**Fig. 2:**
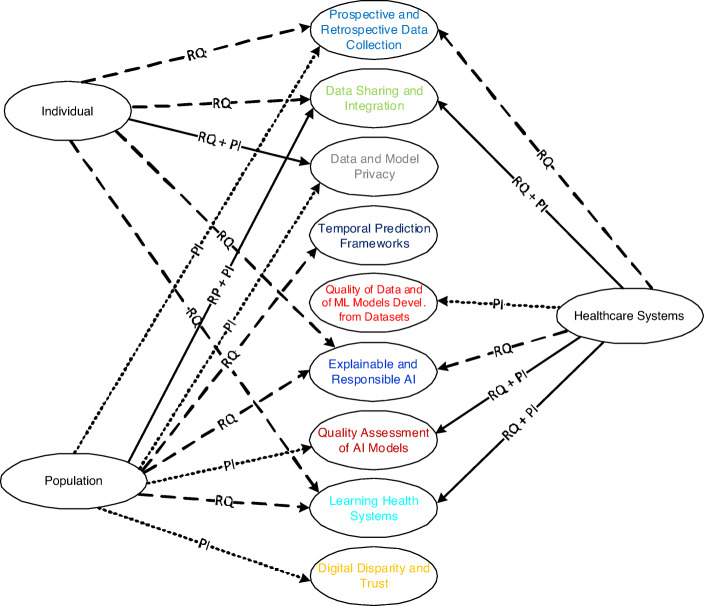
Conceptual map of the research questions (RQ) and of the policy implications (PI) from Table 1. Concepts (ellipses) correspond to views (Individual, Healthcare System, Population) and topics of the research agenda. RQ/PI represent connections between concepts

Table 1 summarizes several major research questions and policy implications, arranged according to the three views. These were also noted at the end of each subsection of Section 3. The same major research questions, policy implications, and views of Table 1 are graphically depicted by Fig. 2. For instance, research topics related to the individual’s view include devising big-data-based methods to answer questions that are important to an individual, even if that individual is not even aware of them. Another example is creating methods for generating explanations, educational materials, and tools that can help a person to make sense of their data. Policy and social implications at the individual’s level regard, for instance, the right of vendors and health actors to access an individual citizen’s data (wearable, behavioral, clinical) to help in improving health services (both private and public) and in preventing or minimizing pandemic effects. The right of an individual to the usability refers to being able to understand the specific functionalities of healthcare services, and the consequences of performing or not performing an action through the digital health system. This can be also considered a policy issue in the individual’s view. At the healthcare level, research methods and tools should be invented for data collection (e.g., involving telemedicine), data aggregation, visualization, and analysis. Regulations at the healthcare level should address, for instance, standards for evaluating ML-based models (e.g., for performing risk calculations). These regulations should go beyond current efforts, such as TRIPOD [97] or SaMD [98].

Methods for semantic data integration and analysis are some of the new research directions. Health policies have to reconsider ethical issues and technological standards for allowing sharing of data in a secure, privacy-conserving, yet meaningful way at the population level.

## Conclusions

In this report, we discuss health informatics issues and related lessons learned from the CoViD-19 pandemic. At the same time, we provide directions for future development of research and the application of the research results in the post-pandemic era, divided into nine themes. The proposed themes of the research agenda are organized into three levels: individual care, healthcare system, and population view, with research topics and their potential policy implications, described for each level. An overarching observation of this group is that, while we recognize that there is substantial room for new informatics innovation, the pandemic demonstrated that there are many mature informatics techniques that are not routinely used in health informatics due to policy and/or public perception arguments, but that proved to be of great value when we operated in crisis mode during the pandemic.

To complete our report, we would like to underline some key points, especially in the context of policy implications and the practical value of the proposed research agenda. One of them is the right to access vendor-specific data and metadata from wearables and sensors of an individual, but also from all online activities of an individual, in a standardized way that allows the data to be analyzed and integrated into the personal EHR . Technical specifications must balance the completeness of established medical standards (e.g., HL7 , FHIR) with the vendors’ needs for lightweight and relatively simple interfaces.

Usable interfaces should be developed for sharing ML models and datasets so that they can be accessed by genetic consultants, radiologists, neurologists, and other professionals. Legislation should be established to define these requirements, as well as the quality of data and ML models developed from datasets. A comprehensive evaluation of ML models, beyond standard ROC and predictive values, merit functions should be required for submissions in scientific literature as well as in the introduction of ML models in clinical practice, with an understanding of the role that end-to-end uncertainty of the individual predictions has to play.

Regulatory frameworks are needed to allow accessing and defining inclusion and exclusion criteria for patients who are similar in characteristics to the patient at the point of care. Acts, technology, and standards for continuity of care should allow clinicians to use tools to obtain evidence-based advice based on AI-supported systems.

On the population level, we identified the need for technological tools to allow sharing of data in a secure, privacy-preserving yet meaningful way (e.g., encryption, de-identification, and blockchain). A step in this direction is represented by the European Health Data Space (EHDS) regulations that offer the possibility or mandate of sharing ML models and data. Another possible solution could include the integration of simulated population data with the same properties as the data of the real organization (i.e., digital twins).

Although this report proposes many new directions and possible solutions to known issues, there are still many open questions that will need to be addressed in the near future. For example, what are the best tools to ensure that data is shared in a secure, privacy-conserving yet meaningful way? Or how can we accelerate the acquisition of data relevant to public health decisions?

Moving to the sensitive argument related to funding strategies and research directions, health informatics investments in terms of research funding models have been strongly influenced by pandemics. For instance, the USA NIH research funding model specific to the pandemic has been defined and used to represent a relevant issue that has to be considered.

A significant limitation of this report that needs to be acknowledged here is that we have not conducted a detailed analysis of the implications of this research agenda on healthcare financial models, a task that will be challenging due to the high variability of these models, even in well-developed countries with advanced healthcare systems.

We conclude with a final issue that will deserve further discussion in the next few years. Indeed, in these years the (public) role of scientists, and that of biomedical and health informatics scientists, has changed. In many situations some colleagues had to manage unexpected overexposure in the mass media, overcoming all the issues related to the communication of scientific content, having a possibly heavy impact also on policy decisions and social behaviors, in a plain and widely understandable language. Such a new role requires specific attention and skills, not completely considered previously, which deserve specific actions also in the way BMHI scientists make their research results accessible and usable worldwide, avoiding misuses and wrong expectations.
